# Mechanisms Challenges of the Pain Phenomenon

**DOI:** 10.3389/fpain.2020.574370

**Published:** 2021-02-10

**Authors:** Serge Marchand

**Affiliations:** Pain Neurophysiology Laboratories, Department of Surgery, Sherbrooke Hospital Research Center, Université de Sherbrooke, Sherbrooke, QC, Canada

**Keywords:** pain, mechanisms, neurophysiology, neuroscience, sex, gender, diffuse noxious inhibitory control (DNIC), conditioned pain modulation (CPM)

Pain is a complex phenomenon involving interconnected physiological and psychological mechanisms. This complexity is a challenge for scientists and clinicians who have to deal with anatomical, physiological, cognitive, and affective pain components.

The nociceptive signal coming from an injury will follow peripheral and central nervous pathways, but will also be modulated by endogenous mechanisms that will increase or reduce the signal and the perceived pain. It is for this reason that we distinguish nociception from pain. Nociception refers to the signal traveling in the nervous system while pain is the perception of the unpleasant experience. Because this signal will be modulated at different levels of the central nervous system, pain is not the mere reflection of nociception. Genetic, environmental, societal, physiological, and psychological factors will influence the perception of pain.

Regardless of the evolution of our knowledge, millions of patients are still suffering and are not good responders to available treatments. We need to refine our understanding of pain mechanisms and the interactions of physiological, psychological, and societal factors.

## The Evolution of Pain Mechanisms Theories

The evolution of our knowledge is driven by hypotheses that scientists are testing that will eventually lead to theories. Challenging new paradigms need to emerge to support new research protocols that will lead to new theories. These theories are the base of our knowledge of pain mechanisms and new treatments. If the theory is based on the wrong assumptions or ones that are only partially true, the resulting treatment development may fail or work for a different reason. Positive results based on the wrong assumption will lead us to adopt incorrect theories more widely and encourage the development of treatment with the same rational that will eventually fail.

Past pain theories were not necessarily wrong but were incomplete, as they did not take into account the complexity of the pain phenomenon. For this reason, it is worth having a brief overview of the evolution of pain theories. It helps us realize that the evolution of pain treatments is highly related to a variety of older and newer pain theories that support our view of pain mechanisms.

### Specificity Theory

The specificity theory was introduced by Descartes during the seventeenth century (1) and was refined with the development of physiology at the end of the nineteenth century (2, 3). The somatosensory system was proposed to be divided into specific receptors for tactile, hot, cold, and pain receptors. The specificity theory is still confirmed by the identification of specific receptors, fibers, pathways, and central nervous system (CNS) structures that are responsible for our perception of these somatosensory modalities, including the nociceptive signal.

There is no doubt that specific pathways are needed to carry the nociceptive signal to the brain. The existence of several pathways, such as from peripheral Ad and C fibers to the spinothalamic tract, or from the spinal cord to the thalamus and to the somatosensory, insular, and cingulate cortices and other regions, has been supported by several electrophysiological and imaging studies. However, even if these pathways exist, specificity is not enough to explain why a stimulus in the same region will become more painful with time (temporal summation) or on a wider surface (spatial summation), or explain complex pain conditions such as phantom pain.

### Pattern Theory

At the end of the nineteenth century, Goldscheider suggested that the stimulation of the same somatosensory receptors could produce a non-painful or a painful perception depending on the pattern of impulses that change according to the intensity or duration (temporal summation) of the stimulus. For instance, a thermal stimulation will be perceived from warm to burning hot if the stimulation persists at the same temperature. The same receptors and pathways are activated, but the frequency of discharge in the neurons will increase and be perceived as more intense.

These nervous patterns help to understand complex phenomena such as allodynia, pain from a non-painful stimulus, or spontaneous pain in conditions where no apparent lesions are detectable. Recent brain imaging and electrophysiological approaches support the importance of activity patterns and not just anatomical locations to render the complexity of pain perception. As an example, there are many studies investigating the synchronous gamma-band frequency (30–100 Hz) as a biomarker of pain perception (4).

### Gate Control Theory

In 1965, the Gate Control Theory was introduced by Melzack and Wall (5). This theory was based on both specificity and pattern theory since it implied that the non-painful Aβ fibers from the periphery would block the nociceptive activity directly at their entrance in the spinal cord, producing a localized hypoalgesia. This theory had an important effect on the development of treatment aimed at stimulation non-nociceptive afferences by peripheral (TENS) or central (DCS) electrical stimulation.

In the same model, Melzack and Wall suggested that descending higher centers' efferences and spinal cord modulations of the nociceptive afferences participated in the experience of pain.

### Diffuse Noxious Inhibitory Controls

A few years after the Gate Control theory, Reynolds demonstrated that stimulation of the periaqueductal gray (PAG) in the brainstem produced a strong inhibition (6). Structures such as the PAG and the nucleus Raphe Magnus (NRM) project serotonergic and noradrenergic to the spinal cord inhibitory interneurons, blocking the nociceptive signal. In the late'70s, Le Bars proposed a model known as diffuse noxious inhibitory controls (DNIC) (7, 8). Based on this model, he demonstrated that a localized nociceptive stimulus could recruit these inhibitory pathways and produce a diffuse hypoalgesia.

## Recent Hypothesis and Theories

Our present understanding of the mechanisms implicated in the development, persistency, and treatment of pain is a combination of previous and more recent theories on pain mechanisms. New theories have emerged.

### Pain Matrix

The “pain matrix,” introduced by Melzack (9), paved the way for studies that identified the structures implicated in different components of the pain experience. Most imaging studies report activity in a number of brain sites, which include sensory (SI, SII), affective (ACC/MCC, insula, PFC), cognitive (ACC/MCC, PFC, SII), and motor (SMA, cerebellum) aspects of pain (10, 11). However, it has been proposed that there is no specific pain matrix since these regions can also be activated by different stimulation modalities and be more related to the salience of the stimuli rather than being specific to pain (12).

### Pain as a Homeostatic Emotion

Rather than seeing pain as part of the exteroceptive sense of touch, Bud Craig suggests that pain is a homeostatic signal (13), based on neuroanatomical and neurophysiological demonstrations. Pain perception is then both a distinct sensation and a motivation at the same time. The earliest brain activity following a nociceptive stimulus is in the posterior insula and mid-cingulate cortex (14), two regions that play a role in affective reactions and in homeostasis.

### Interaction of Endogenous Excitatory and Inhibitory Mechanisms

Persistent pain can result from the recruitment of excitatory mechanisms, such as central sensitization or the reduction of the efficacy of inhibitory mechanisms (15, 16). Even if two patients present apparently similar pain conditions, the mechanisms may be different and will not respond to the same treatments. For example, for excitatory hyperactivity (central sensitization), anticonvulsant may be the treatment of choice. But, if a deficit of inhibitory mechanisms is implicated, antidepressants to trigger back serotonergic and noradrenergic endogenous inhibitory mechanisms may be a better choice (17).

These results support that new approaches to measure endogenous pain modulation mechanisms in chronic pain will help to guide treatment. Different brain imaging techniques are part of the tools that will help identify specific mechanisms and the specific effects of some treatments (11, 18).

### Genetic Factors

The relation between nociceptive activity and pain perception is influenced by inherited and external influences. Studies support a genetic predisposition to be more or less sensitive to pain (19). However, studies with identical twins demonstrate that between 25 and 60% of similarities in the responses depend on the type of experimental pain used, suggesting both inherited and external factors at play (20). The effect of the environment on genetics—epigenetics—is also an important aspect that will influence individualized pain responses (21). Nerve injuries or even psychological factors may have an effect on the central nervous system by disturbing DNA methylation and produce a genomic memory of pain in the cortex (22). It could explain the comorbidity between some psychiatric problems such as depression and anxiety with pain (23). These results support the importance of psychological factors such as mood, anxiety, catastrophizing, and personality in pain perception (24).

## Pain Mechanism Challenges

From classical pain mechanism theories, such as specificity and patterns theories, modern approaches using psychophysics, electrophysiology, biomarkers, and brain imaging have allowed us to refine our understanding of different aspects of pain, from nociceptive activities to chronic pain conditions. However, even if the evolution of our knowledge has been important in recent decades, patients are still suffering from chronic pain conditions that are not responding to available treatments. Moreover, two patients with apparently similar pain conditions will not respond to the same treatments. We can only conclude that our understanding of the complexity of pain is still incomplete and that we need more translational research, from fundamental studies to clinical applications and vice versa.

We also need trans-sectoral approaches, encouraging collaborations not just between experts in health science and medicine, but also between experts from fields such as engineering, computer science, education, or philosophy with physiologists and psychologists. The challenge is to be open-minded and to accept to be challenged by people that are not within our field. We see more research teams that have specialists in Artificial Intelligence (AI), sociology, or biomechanics and patient partners when developing new research protocols. I am convinced that this way of thinking will lead to new paradigms, helping us to better understand how we can embrace the diversity of pain patients and their unique response to treatments.

## Examples of the Domains That Need More Research

### Peripheral Mechanisms Nourishing Chronic Pain

Most of the studies on chronic pain associate chronic pain with a central sensitization, neglecting the role of peripheral mechanisms. Peripheral nociceptor sensitization may play a major role in some chronic pain conditions, including widespread pain conditions (25). The mechanisms related to this maintained sensitization by peripheral activity need to be understood to recognize the importance of looking for peripheral implications in chronic conditions that may appear as a purely central effect. The diagnostics and treatments will be affected by a better understanding of this link.

### Personalized Pain Treatments

All treatments, pharmacological or not, will never provide the same level of relief for every patient, emphasizing a need for personalized treatments. Several neurophysiological and psychological factors are implicated in the mechanisms supporting these differences. We need more research that will help identify the factors responsible for these individual differences. We need more tools to measure them in laboratories, but also more clinical diagnostic protocols to be used in the clinic.

### Sex, Gender, and Pain

Major differences between sex and gender in pain have been documented in both fundamental and clinical research (26, 27). Better understanding pain mechanisms implies taking into account the importance of both biological sex and gender identity.

### Children and Elderly

Age is a critical factor that has been neglected for too long in understanding pain mechanisms. Children (28) and the elderly (29) are two populations with specific pain problems and specific responses to treatment. More translational research to better characterize the mechanisms in these populations is needed to take care of all the populations suffering from pain conditions.

### Mental Health and Pain

The link between mental health and pain is a major topic (30). We know that mental health conditions, such as depression or anxiety, are important factors in the development and persistency of pain, even for post-operative pain (31). Moreover, there is an overlap in the pharmacological treatment of mental health and chronic pain (32). The mechanism linking mental health and pain is a very important topic of research for developing better approaches to pain treatment.

### Similitude and Differences in Preclinical and Clinical Research

Too often, a new pharmacological target will be found to be a potent analgesic in animal models but does not work well in humans (33). Translational research between animal models and humans needs to go in both directions, in order to guarantee the use of proper models for future research.

### Endogenous Pain Modulation: Science and Clinical Applications

Deficits in endogenous excitatory and inhibitory mechanisms seem to play an important role in several chronic pain conditions. More research is needed to translate these findings into the clinic. We need simple and appropriate tools to measure these mechanisms in patients and standardized response profiles in healthy subjects and patients suffering from chronic pain for clinical tools that will help in the development of personalized pain treatments.

## Conclusion

Pain is an important alarm that encourages us to avoid an injury or to protect the injured body part during the healing process. However, too often the alarm role is overpassed, and chronic pain conditions greatly impact the quality of life of the person suffering. The search to reduce pain has been a long journey that has been documented in old medical texts. It is interesting to realize to what point the proposed treatments are based on the theories of a specific time. “Bloodletting” was accepted as a good treatment when pain was perceived as an “intruder” that we wanted to chase from the body. It is likely that bloodletting worked well since the cutting was painful, producing a counter-irritation analgesia and strong placebo effects, reinforcing the theory that something in the blood needed to be released.

Modern theories focused on the nervous system, from the periphery to the higher center, for pain. Patterns theories gave us the opportunity to realize that nervous system structures and nervous system activity modulation are responsible for our perception of pain.

The goal of this journal is to encourage the publication of great works on pain mechanisms. Challenging old and current theories will help retain what seems to make sense and introduce new pieces of information to help comprehend pain, and thus become more efficient in controlling it in the largest percentage of patients possible.

## Author Contributions

The author confirms being the sole contributor of this work and has approved it for publication.

## Conflict of Interest

The author declares that the research was conducted in the absence of any commercial or financial relationships that could be construed as a potential conflict of interest.
